# Supplementary material for: SLD5/GINS4 controls dynein-dependent centrosome maturation and exposes a candidate mitotic vulnerability in cancer
Source: bioRxiv. 2026 May 11:2026.05.07.723511. Preprint. [Version 1] doi: 10.64898/2026.05.07.723511 (PMC13192919; doi:10.64898/2026.05.07.723511)
Supplement: 1 [file NIHPP2026.05.07.723511v1-supplement-1.pdf]

bioRxiv preprint doi: https://doi.org/10.64898/2026.05.07.723511; this version posted May 11, 2026. The copyright holder for this preprint (which was not certified by peer review) is the author/funder, who has granted bioRxiv a license to display the preprint in perpetuity. It is made available under a CC-BY 4.0 International license.

Supplementary figure legends

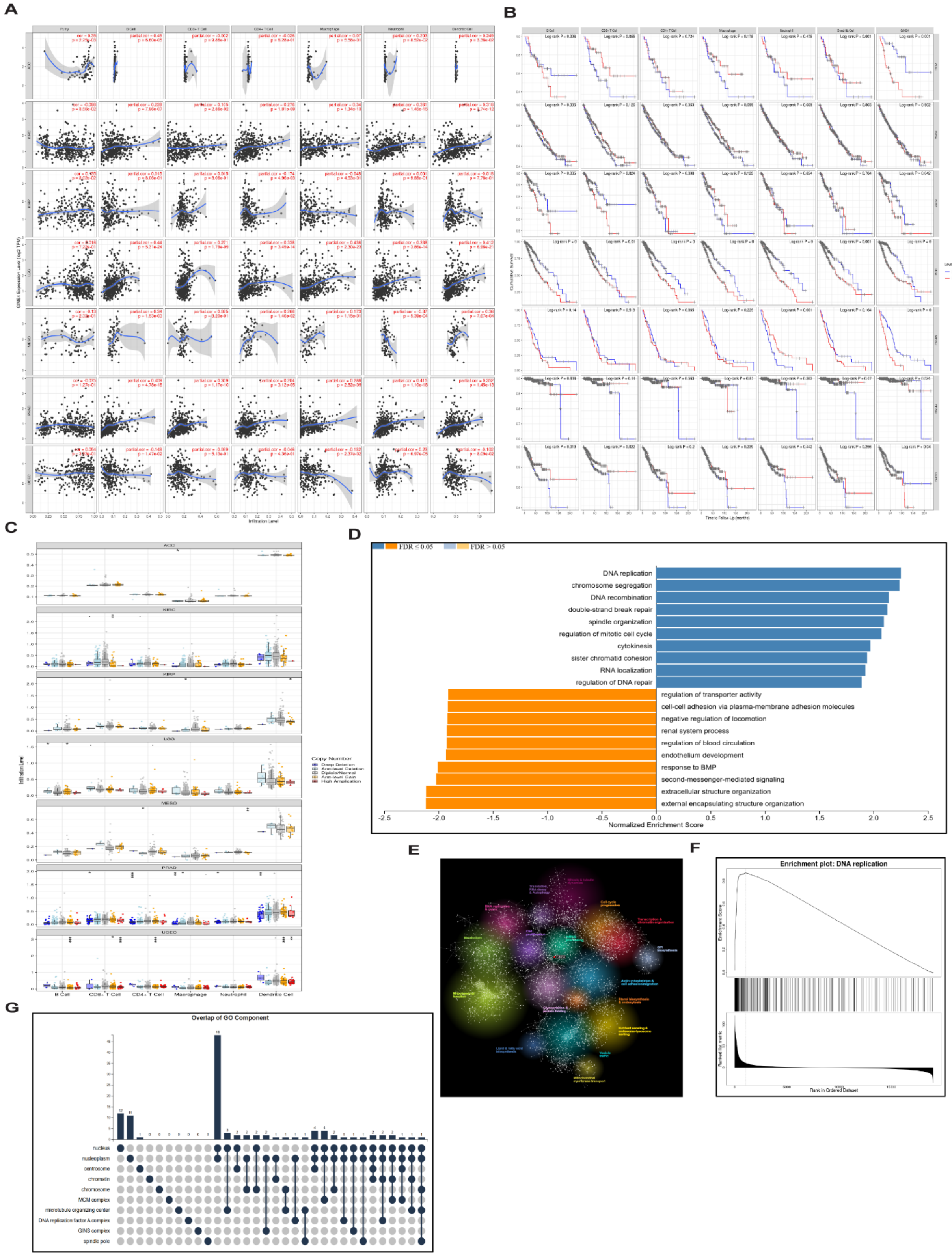

bioRxiv preprint doi: https://doi.org/10.64898/2026.05.07.723511; this version posted May 11, 2026. The copyright holder for this preprint (which was not certified by peer review) is the author/funder, who has granted bioRxiv a license to display the preprint in perpetuity. It is made available under a CC-BY 4.0 International license.

Supplementary Fig. S1. Immune-context and pathway analyses of GINS4-associated tumor states.

A. Correlation analysis between GINS4 expression and tumor purity or immune-cell infiltration across selected cancer types. Immune compartments include B cells, $CD8^+$ T cells, $CD4^+$ T cells, macrophages, neutrophils, and dendritic cells. Correlation coefficients and P values are shown within each panel.

B. Kaplan–Meier survival analyses stratified by high versus low GINS4 expression or immune-infiltration estimates across selected tumor contexts. Log-rank P values are shown in each panel.

C. Association between GINS4 copy-number status and immune infiltration levels across selected cancer types. Copy-number categories include deep deletion, arm-level deletion, diploid/normal, arm-level gain, and high amplification. Box plots show the distribution of immune-infiltration estimates within each copy-number group.

D. Gene set enrichment analysis of GINS4-associated transcriptional programs. Bars indicate normalized enrichment scores. Positively enriched pathways include DNA replication, chromosome segregation, DNA recombination, double-strand break repair, spindle organization, mitotic cell-cycle regulation, cytokinesis, and DNA repair regulation. Color denotes FDR status as indicated in the plot.

E. Enrichment-map visualization of functionally related gene sets associated with GINS4-linked transcriptional programs. Gene-set clusters highlight biological processes associated with replication, mitosis, chromosomes, and DNA repair.

F. Running enrichment plot for the DNA replication gene set, showing enrichment of DNA replication genes among the GINS4-associated ranked gene list.

G. UpSet plot showing overlap among Gene Ontology cellular-component annotations for GINS4-associated genes. Enriched components include the nucleus, nucleoplasm, centrosome, chromatin, chromosome, MCM complex, microtubule-organizing center, DNA replication factor A complex, GINS complex, and spindle pole.

bioRxiv preprint doi: https://doi.org/10.64898/2026.05.07.723511; this version posted May 11, 2026. The copyright holder for this preprint (which was not certified by peer review) is the author/funder, who has granted bioRxiv a license to display the preprint in perpetuity. It is made available under a CC-BY 4.0 International license.

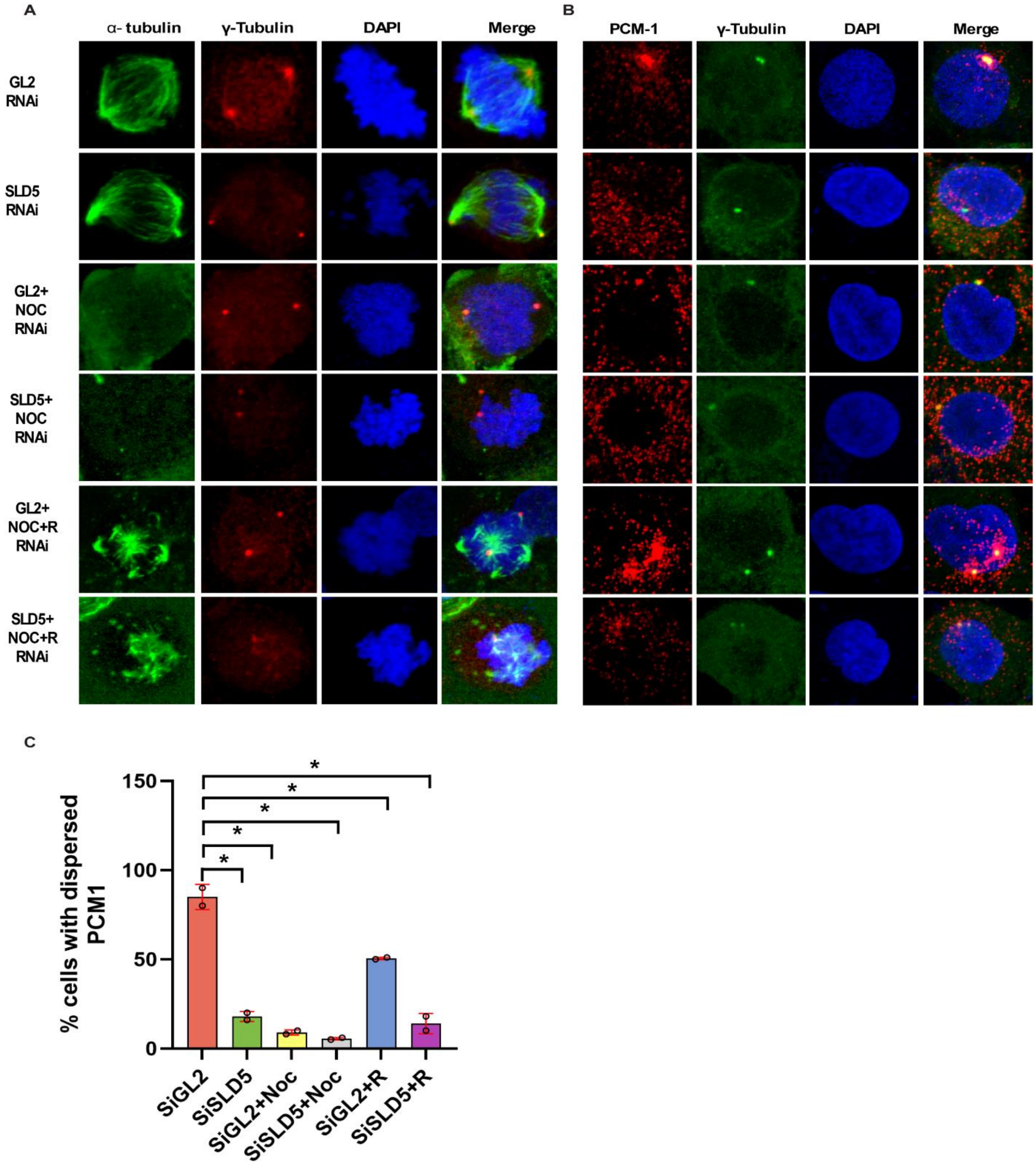

Supplementary Fig. S2. Sld5 depletion impairs centriolar satellite reassembly after microtubule regrowth.

A. Representative immunofluorescence images showing α-tubulin, γ-tubulin, and DAPI staining in GL2 control and Sld5-depleted cells under basal conditions, after nocodazole treatment, and after nocodazole washout/recovery. Nocodazole disrupts the microtubule network, whereas recovery allows microtubule regrowth.

bioRxiv preprint doi: https://doi.org/10.64898/2026.05.07.723511; this version posted May 11, 2026. The copyright holder for this preprint (which was not certified by peer review) is the author/funder, who has granted bioRxiv a license to display the preprint in perpetuity. It is made available under a CC-BY 4.0 International license.

B. Representative immunofluorescence images showing PCM1, γ-tubulin, and DAPI staining under the same conditions as in a. PCM1 is organized around centrosomes in GL2 control cells, becomes dispersed after nocodazole-induced microtubule depolymerization, and reassembles after microtubule recovery. In Sld5-depleted cells, PCM1 remains dispersed despite microtubule regrowth.

C. Quantification of cells with dispersed PCM1 signal in GL2 control and Sld5-depleted cells under basal, nocodazole-treated, and recovery conditions. NOC denotes nocodazole treatment; R denotes recovery after nocodazole washout. Dots indicate individual measurements; error bars indicate variation among measurements; *$P < 0.05$.

bioRxiv preprint doi: https://doi.org/10.64898/2026.05.07.723511; this version posted May 11, 2026. The copyright holder for this preprint (which was not certified by peer review) is the author/funder, who has granted bioRxiv a license to display the preprint in perpetuity. It is made available under a CC-BY 4.0 International license.

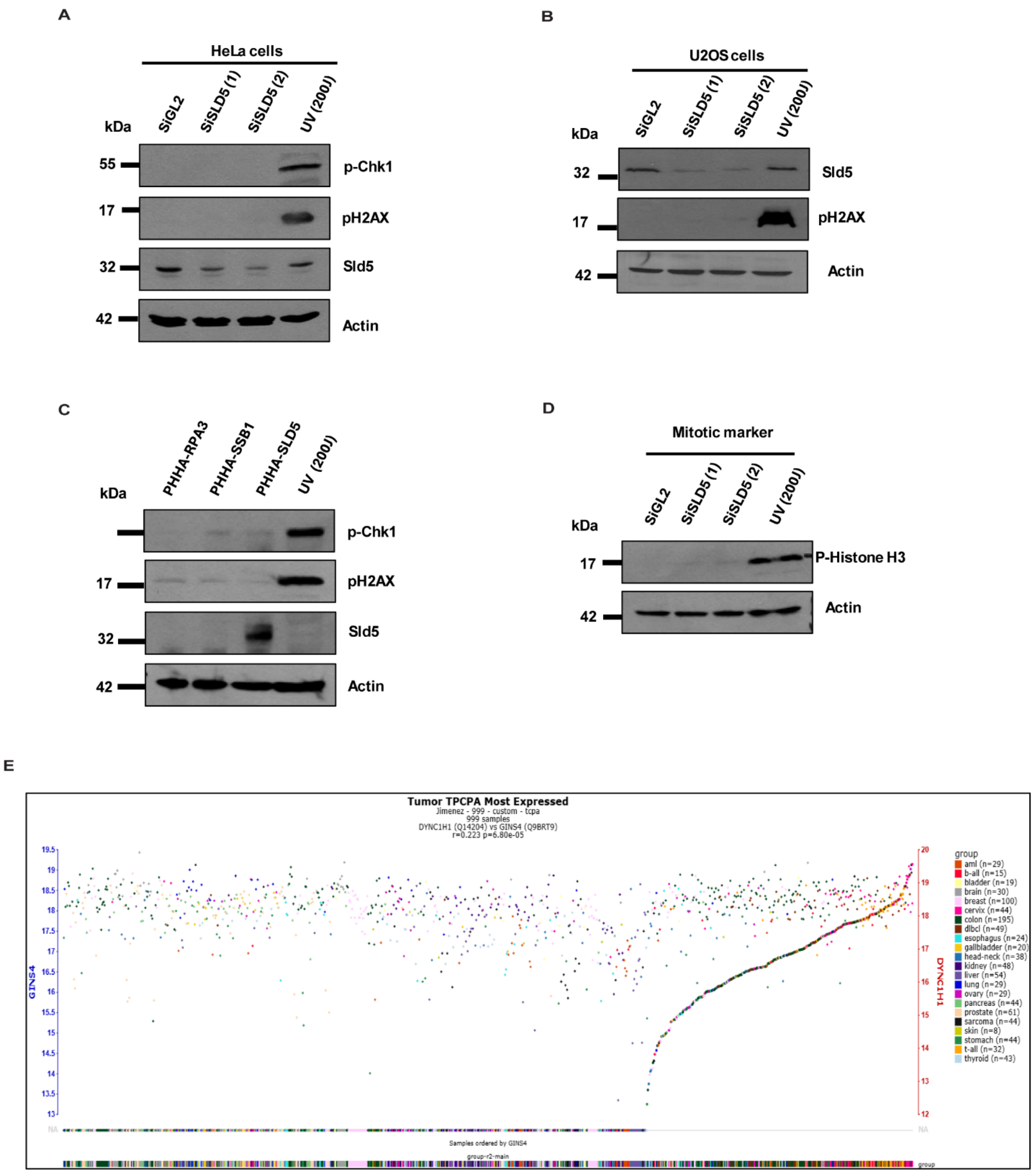

Supplementary Fig. S3. Sld5 depletion or overexpression does not induce detectable DNA damage signaling.

A. Immunoblot analysis of phospho-Chk1, phospho-H2AX, Sld5, and actin in HeLa cells after GL2 control RNAi or Sld5 depletion using two independent RNAi reagents. UV-treated cells were used as a positive control for DNA damage induction. Sld5 depletion does not induce detectable phospho-Chk1 or phospho-H2AX accumulation.

bioRxiv preprint doi: https://doi.org/10.64898/2026.05.07.723511; this version posted May 11, 2026. The copyright holder for this preprint (which was not certified by peer review) is the author/funder, who has granted bioRxiv a license to display the preprint in perpetuity. It is made available under a CC-BY 4.0 International license.

B. Immunoblot analysis of Sld5, phospho-H2AX, and actin in U2OS cells after GL2 control RNAi or Sld5 depletion. UV-treated cells were used as a positive control. Sld5 depletion does not induce detectable phospho-H2AX accumulation.

C. Immunoblot analysis of phospho-Chk1, phospho-H2AX, Sld5, and actin in cells overexpressing Sld5 or control constructs. UV-treated cells served as a positive control. Sld5 overexpression does not induce detectable DNA damage signaling.

D. Immunoblot analysis of phospho-histone H3 in GL2 control and Sld5-depleted cells. Actin was used as a loading control. Sld5 depletion does not cause a marked increase in phospho-histone H3 under these conditions.

E. Tumor proteomic analysis showing the association between GINS4/SLD5 and DYNC1H1 protein abundance across tumor samples. Samples are ordered by GINS4 expression and coloured by tumor group, supporting tumor-context-dependent co-variation between GINS4/SLD5 and DYNC1H1.

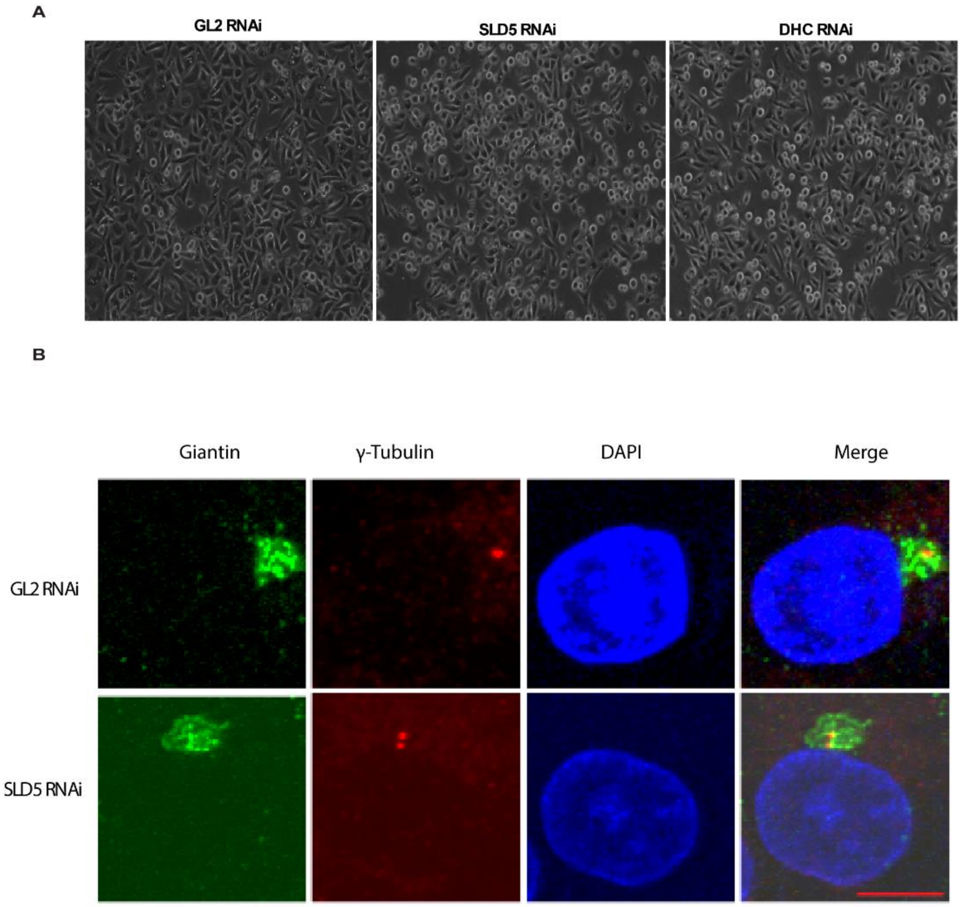

Supplementary Fig. S4. Sld5 depletion does not broadly disrupt Golgi organization.

bioRxiv preprint doi: https://doi.org/10.64898/2026.05.07.723511; this version posted May 11, 2026. The copyright holder for this preprint (which was not certified by peer review) is the author/funder, who has granted bioRxiv a license to display the preprint in perpetuity. It is made available under a CC-BY 4.0 International license.

A. Representative phase-contrast images of GL2 control, Sld5-depleted, and DHC-depleted cells. Sld5 and DHC depletion produce comparable cellular perturbation, consistent with overlapping effects on mitotic and centrosome-associated processes.

B. Representative immunofluorescence images showing localization of the Golgi marker giantin in GL2 control and Sld5-depleted cells. Cells were stained for giantin, γ-tubulin, and DAPI. Giantin retains a compact perinuclear Golgi-like distribution after Sld5 depletion, indicating that Sld5 loss does not cause a generalized disruption of organelle organization.

bioRxiv preprint doi: https://doi.org/10.64898/2026.05.07.723511; this version posted May 11, 2026. The copyright holder for this preprint (which was not certified by peer review) is the author/funder, who has granted bioRxiv a license to display the preprint in perpetuity. It is made available under a CC-BY 4.0 International license.

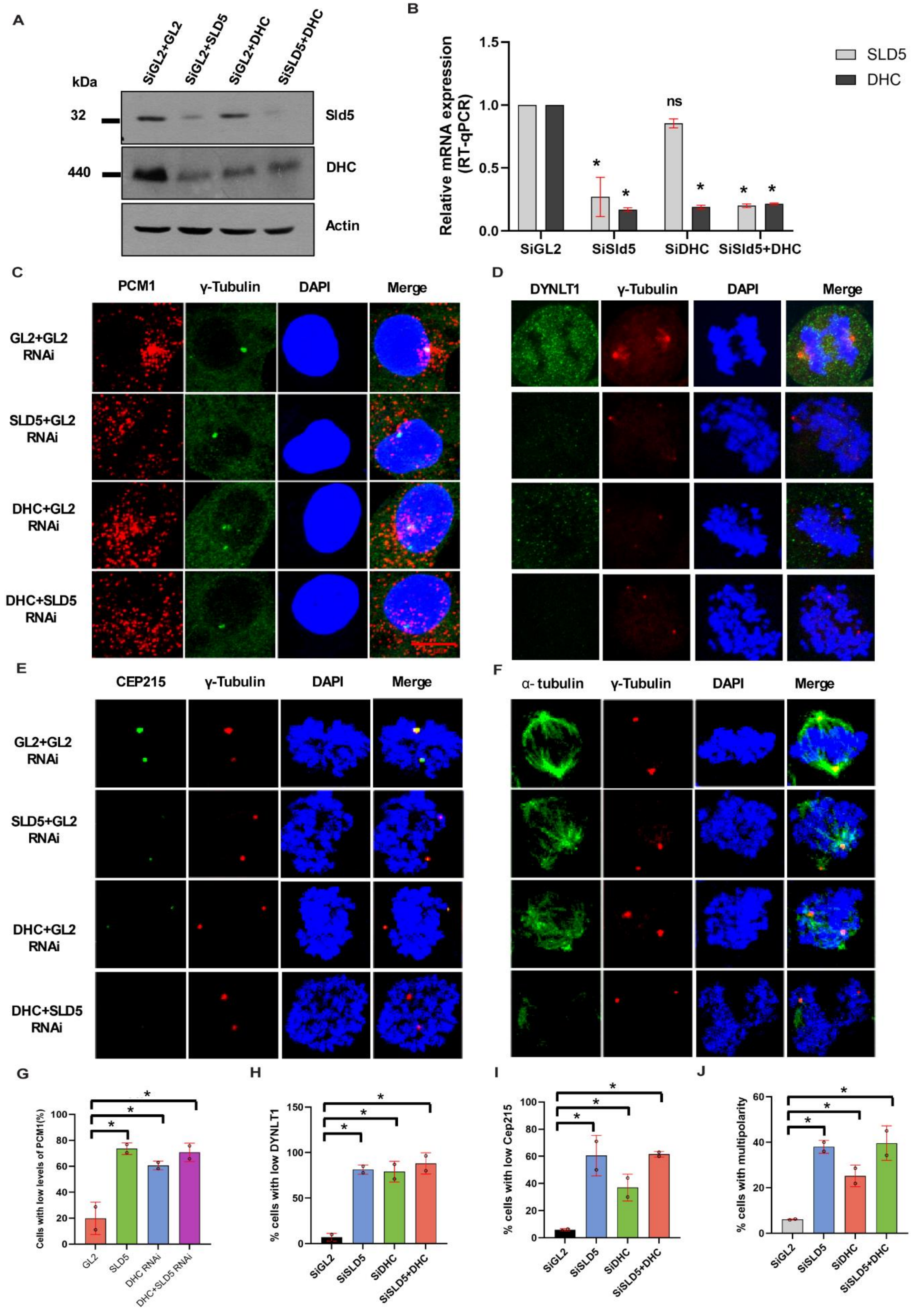

bioRxiv preprint doi: https://doi.org/10.64898/2026.05.07.723511; this version posted May 11, 2026. The copyright holder for this preprint (which was not certified by peer review) is the author/funder, who has granted bioRxiv a license to display the preprint in perpetuity. It is made available under a CC-BY 4.0 International license.

Supplementary Fig. S5. Co-depletion of Sld5 and DHC does not exacerbate centrosome–satellite defects, supporting a shared pathway.

A. Immunoblot analysis of Sld5 and DHC protein levels after GL2 control RNAi, Sld5 RNAi, DHC RNAi, or combined Sld5 and DHC RNAi. Actin was used as a loading control.

B. RT–qPCR analysis of SLD5 and DHC transcript levels after individual or combined depletion. Sld5 and DHC depletion is confirmed at the mRNA level. Bars show relative expression; error bars indicate variation among measurements. ns, not significant; *P < 0.05.

C. Representative immunofluorescence images showing PCM1 localization after control, Sld5, DHC, or combined Sld5 and DHC RNAi. Cells were stained for PCM1, γ-tubulin, and DAPI. PCM1 is dispersed after Sld5 or DHC depletion, and combined depletion does not further increase the phenotype.

D. Representative immunofluorescence images showing DYNLT1 localization after control, Sld5, DHC, or combined Sld5 and DHC RNAi. Cells were stained for DYNLT1, γ-tubulin, and DAPI. DYNLT1 signal is reduced after Sld5 or DHC depletion, with no clear additive effect after co-depletion.

E. Representative immunofluorescence images showing CEP215 localization after control, Sld5, DHC, or combined Sld5 and DHC RNAi. Cells were stained for CEP215, γ-tubulin, and DAPI. CEP215 recruitment to centrosomes is reduced after Sld5 or DHC depletion.

F. Representative immunofluorescence images showing spindle organization after control, Sld5, DHC, or combined Sld5 and DHC RNAi. Cells were stained for α-tubulin, γ-tubulin, and DAPI. Sld5 and DHC depletion produce spindle-pole defects, and combined depletion does not markedly exacerbate the phenotype.

G. Quantification of cells with low PCM1 signal after individual or combined Sld5 and DHC depletion.

H. Quantification of cells with low DYNLT1 signal after individual or combined Sld5 and DHC depletion.

I. Quantification of cells with low CEP215 signal after individual or combined Sld5 and DHC depletion.

J. Quantification of cells with multipolar spindles after individual or combined Sld5 and DHC depletion. Co-depletion does not yield an additive increase in multipolarity relative to the strongest single-depletion condition, supporting a shared Sld5–DHC pathway.

Supplementary Table legends

Supplementary Table S1. Antibodies list for immunofluorescence.

Supplementary Table S2. Antibodies list for immunoprecipitation.

Supplementary Table S3. Antibodies list for western blot.

bioRxiv preprint doi: https://doi.org/10.64898/2026.05.07.723511; this version posted May 11, 2026. The copyright holder for this preprint (which was not certified by peer review) is the author/funder, who has granted bioRxiv a license to display the preprint in perpetuity. It is made available under a CC-BY 4.0 International license.

Supplementary Table S4. Genes primer list for RT-qPCR.

Supplementary Table S5. Genome-wide Stouffer's Z-score ranking from the integrated pan-cancer analysis.

This table reports gene-level Stouffer's Z-scores generated from the integrated pan-cancer analysis. Genes are ranked by their combined Z-score, with positive values indicating enrichment in the positive pan-cancer signal and negative values indicating enrichment in the opposite direction. GINS4 is listed within the positive tail of the distribution with a Stouffer's Z-score of 9.1413. Columns include gene symbol and Stouffer's Z-score.

Supplementary Table S5A. H-score quantification of GINS4 immunohistochemical staining in representative normal and tumor tissues. This table summarizes semi-quantitative H-score analysis of GINS4 immunohistochemistry in representative normal and tumor tissue sections. Normalized tumor staining was calculated relative to the indicated normal comparator. Liver cholangiocarcinoma showed a 2.67-fold increase in GINS4 staining compared with normal liver/hepatocytes, whereas prostate adenocarcinoma showed a 1.96-fold increase compared with the indicated normal comparator tissue. Columns include comparison, normal tissue, normal H-score, cancer tissue, cancer H-score, normalized normal value, normalized cancer value, and fold change.

Supplementary Table S6. Cancer-lineage-specific association between SLD5 knockout effect and DYNC1H1 expression. This table summarizes correlation analyses between SLD5 knockout-associated log fold-change and DYNC1H1 expression across cancer types. For each lineage, Pearson and Spearman correlations, coefficient of determination, regression slope, and regression intercept are reported. The analysis identifies tumor contexts with inverse or positive relationships between SLD5 loss and DYNC1H1 expression, supporting cancer-type-specific regulation of the SLD5–dynein heavy-chain axis.

Supplementary Table S7. Cancer-lineage-specific association between SLD5 overexpression and DYNC1H1 expression. This table summarizes correlation analyses between SLD5 expression in SLD5-overexpressing tumor contexts and DYNC1H1 expression across cancer types. For each lineage, Pearson and Spearman correlations, coefficient of determination, regression slope, and regression intercept are reported. Positive associations in several carcinoma lineages support coordinated regulation of GINS4/SLD5 and DYNC1H1 in selected tumor contexts, whereas weaker or inverse correlations indicate lineage-specific differences in this relationship.

Supplementary Table S8. Pan-cancer correlation analysis of GINS4 and POLR2A expression. This table summarizes cancer-lineage-specific correlations between GINS4/SLD5 and POLR2A expression. For each cancer type, Pearson and Spearman correlations, coefficient of determination, regression slope, and regression intercept are reported. Positive

bioRxiv preprint doi: https://doi.org/10.64898/2026.05.07.723511; this version posted May 11, 2026. The copyright holder for this preprint (which was not certified by peer review) is the author/funder, who has granted bioRxiv a license to display the preprint in perpetuity. It is made available under a CC-BY 4.0 International license.

correlations across multiple tumor lineages support coordinated regulation of GINS4 and POLR2A, suggesting a potential connection between Sld5 and RNA polymerase II-associated transcriptional activity in cancer.

Supplementary Table S9. Predicted kinase associations with GINS4/SLD5. This table lists candidate kinases predicted to interact functionally with GINS4/SLD5 or to be associated with it. Genes are ranked by Z-score, with higher Z-scores indicating stronger predicted association. The top-ranked candidates include BUB1, WEE1, CDC7, EIF2AK1, VRK2, NME2, STK16, NEK2, TAF1, PLK4, TTK, and PLK1, highlighting mitotic checkpoint, centrosome maturation, and replication-associated kinase pathways as potential therapeutic nodes in SLD5-dependent tumors.

Supplementary Table S10. Co-occurrence analysis of GINS4/SLD5-associated kinase candidates. This table summarizes co-occurrence relationships among GINS4/SLD5 and candidate kinase genes. Columns report the number of cases with neither alteration, alteration of A only, alteration of B only, alteration of both genes, $\log_2$ odds ratio, P value, q value, and co-occurrence tendency. Significant co-occurrence was observed among several kinase pairs, including GINS4–EIF2AK1, GINS4–WEE1, and GINS4–PLK1, supporting a coordinated kinase network linked to GINS4/SLD5-associated cancer dependency.
